# Coastal water bacteriophages infect various sets of *Vibrio parahaemolyticus* sequence types

**DOI:** 10.3389/fmicb.2022.1041942

**Published:** 2022-12-19

**Authors:** Kari A. Brossard Stoos, Jennifer Ren, Robin R. Shields-Cutler, Kelly L. Sams, Shannon Caldwell, Marvin B. Ho, Gregg Rivara, Cheryl A. Whistler, Stephen H. Jones, Martin Wiedmann, Jamie DeMent, Rodman G. Getchell, Hélène Marquis

**Affiliations:** ^1^Department of Microbiology and Immunology, Cornell University, Ithaca, NY, United States; ^2^Department of Health Promotion and Physical Education, Ithaca College, Ithaca, NY, United States; ^3^Department of Biology, Macalester College, Saint Paul, MN, United States; ^4^Cornell Cooperative Extension of Suffolk County, Southold, NY, United States; ^5^Northeast Center for Vibrio Disease and Ecology, University of New Hampshire, Durham, NH, United States; ^6^Department of Molecular, Cellular, and Biomedical Sciences, University of New Hampshire, Durham, NH, United States; ^7^Department of Natural Resources and the Environment, University of New Hampshire, Durham, NH, United States; ^8^Department of Food Science, Cornell University, Ithaca, NY, United States; ^9^Florida Department of Health, Tallahassee, FL, United States

**Keywords:** *Vibrio parahaemolyticus*, vibriophage, sequence type, phage, ST36

## Abstract

**Introduction:**

Gastrointestinal illnesses associated with the consumption of shellfish contaminated with *Vibrio parahaemolyticus* have a negative impact on the shellfish industry due to recalls and loss of consumer confidence in products. This bacterial pathogen is very diverse and specific sequence types (STs), ST631 and ST36, have emerged as prevalent causes of *Vibrio* foodborne disease outbreaks in the US, though other STs have been implicated in sporadic cases. We investigated whether bacteriophages could be used as a proxy to monitor for the presence of distinct *V. parahaemolyticus* STs in coastal waters.

**Methods:**

For this purpose, bacteriophages infecting *V. parahaemolyticus* were isolated from water samples collected on the Northeast Atlantic coast. The isolated phages were tested against a collection of 29 *V. parahaemolyticus* isolates representing 18 STs, including six clonal complexes (CC). Four distinct phages were identified based on their ability to infect different sets of *V. parahaemolyticus* isolates.

**Results and Discussion:**

Overall, the 29 bacterial isolates segregated into one of eight patterns of susceptibility, ranging from resistance to all four phages to susceptibility to any number of phages. STs represented by more than one bacterial isolate segregated within the same pattern of susceptibility except for one *V. parahaemolyticus* ST. Other patterns of susceptibility included exclusively clinical isolates represented by distinct STs. Overall, this study suggests that phages populating coastal waters could be exploited to monitor for the presence of *V. parahaemolyticus* STs known to cause foodborne outbreaks.

## Introduction

Gastrointestinal illnesses associated with the consumption of shellfish contaminated with pathogenic *Vibrio parahaemolyticus* have increased in prevalence in recent decades (Haendiges et al., 2014; Newton et al., 2014; Xu et al., 2015; Baker-Austin et al., 2016; Abanto et al., 2020). Between 2009 and 2020, the CDC National Outbreak Reporting System (NORS) documented 151 outbreaks related to *Vibrio* contaminated food in the United States.^1^ In 2014 alone, it was estimated that *V. parahaemolyticus* accounted for over 92,000 individual infections in the United States (Collier et al., 2021). The association of foodborne infections with contaminated shellfish has a significant negative impact not only on public health, but also on the industry due to recalls and, importantly, a loss of consumer confidence in the product. Therefore, there is a need to develop enhanced measures to monitor coastal waters surrounding aquaculture farms for the presence of *V. parahaemolyticus* associated with food infection.

*Vibrio parahaemolyticus* is ubiquitous in coastal waters and bacterial growth increases with an increase in water temperature during the summer months (Ellis et al., 2012; Rodgers et al., 2014). Shellfish, such as oysters and clams, filter large amounts of water to acquire nutrients; consequently, shellfish are more susceptible to contamination with *V. parahaemolyticus* as water temperatures increase during the summer months (Ellis et al., 2012; Rodgers et al., 2014). The National Shellfish Sanitation Program tightly regulates the shellfish industry, and has developed measures to decrease the incidence of contamination from harvest to table.^2^ Diagnostic tests for detection of *V. parahaemolyticus* in seafood exist, e.g., detection of toxin genes by PCR and bacterial isolation. However, results from these tests do not distinguish strains that are associated with gastrointestinal infections in humans from those that do not. *Vibrio parahaemolyticus* is a genetically diverse bacterial species represented by multiple sequence types (STs), some of which are grouped within clonal complexes (CC; Mahoney et al., 2010; Schuster et al., 2011; Ellis et al., 2012; Whistler et al., 2015; Xu et al., 2015, 2017a,b). The large majority of *V. parahaemolyticus* strains are avirulent and no known absolute common genetic features exist among virulent strains (Ronholm et al., 2015). However, specific *V. parahaemolyticus* STs and CCs have been associated with foodborne illnesses. For example, ST3, ST36, ST631 have been the main STs associated with outbreaks. About 25 years ago, infectious *V. parahaemolyticus* strains belonging to CC3, which includes ST3, emerged in India and rapidly disseminated worldwide (Nair et al., 2007). ST3 is still abundant in Asia (Tan et al., 2021), but strains belonging to ST36 and ST631 have been the prevalent sources of foodborne infections in the United States in recent decades (Haendiges et al., 2014; Xu et al., 2015, 2017a,b). ST36 was initially isolated from the Pacific Northwest, and subsequently spread to the Atlantic Northeast. *Vibrio parahaemolyticus* ST36 caused an outbreak in Spain in 2012 and was identified as a source of infection in Peru between 2012 and 2016 (Abanto et al., 2020). In addition, some STs have been associated with sporadic clinical cases (Miller et al., 2021).

Bacteriophages that infect *Vibrio* spp. including *V. parahaemolyticus* have been previously isolated from both seafood and water samples (Wang et al., 2017; Chen et al., 2019; Richards et al., 2019; Le et al., 2020; Tan et al., 2021). In the present study, we assessed whether bacteriophages that populate coastal waters surrounding oyster farms could be used to assess for the presence of specific *V. parahaemolyticus* STs. Four *de novo* isolated bacteriophages were selected based on their ability to infect distinct sets of *V. parahaemolyticus* isolates. The 29 *V. parahaemolyticus* isolates, representing 18 STs, segregated into eight phage susceptibility patterns, each composed of up to four distinct STs. The potential applications of monitoring phage populations in coastal waters to assess for the presence of *V. parahaemolyticus* STs are considered in the discussion.

## Materials and methods

### Bacterial strains and growth conditions

Twenty-nine isolates of *V. parahaemolyticus* were obtained from three different labs (Table 1). This array of strains was comprised of clinical, environmental, and seafood isolates, many of which have been previously characterized and include a broad representation of isolation dates and STs. *Escherichia coli*, *Bacillus subtilis*, *Pseudomonas fluorescens*, *Pseudomonas aeruginosa*, *Edwardsiella piscicida*, *Vibrio cholerae*, and *Aeromonas hydrophila* were included in the study to demonstrate species specificity to bacteriophage infection. Bacteria were grown in LB Lennox (LBL), Tryptic Soy Broth (TSB) or, on Tryptic Soy Agar (TSA) supplemented with 2% NaCl. Isolates were stored at −80°C in 50%glycerol/50%TSB + 2%NaCl.

**Table 1:** *Vibrio parahaemolyticus* isolates used in this study.

Strain ID	Source	Place of isolation	Year of isolation	Sequence type	Clonal complex
FSL Y1-003^a^	Clinical	Japan	Unknown	88	345
FSL Y1-005^a^	Food (oyster)	WA, United States	1988	8	8
FSL Y1-010^a^	Clinical	Japan	Unknown	326	None
FSL Y1-012^a^^,^^e^	Clinical	WA, United States	1991	1748	None
FSL Y1-013^a^	Clinical	WA, United States	1991	54	None
FSL Y1-015^a^	Clinical	Bangladesh	1998	3	3
FSL Y1-016^a^	Clinical	TX, United States	1998	3	3
FSL Y1-017^a^^,^^e^	Clinical	NY, United States	1998	1464	3
FSL Y1-021^a^	Clinical	Bangladesh	1980	87	None
FSL Y1-023^a^	Clinical	India	1996	3	3
FSL Y1-024^a^	Clinical	India	1997	3	3
FSL Y1-025^a^	Clinical	India	1997	3	3
FSL Y1-026^a^	Clinical	India	1996	3	3
FSL Y1-036^a^^,^^e^	Clinical	Unknown	Unknown	3	3
FSL Y1-046^a^	Clinical	NY, United States	Unknown	3	3
FSL Y1-059^a^	Food (oyster)	TX, United States	1998	676	None
FSL Y1-068^a^	Food (oyster)	AL, United States	Unknown	54	None
FSL Y1-069^a^	Food (oyster)	AL, United States	Unknown	26	24
FSL Y1-078^a^^,^^e^	Unknown	Unknown	Unknown	46	None
FSL Y1-079^a^	Unknown	Unknown	Unknown	46	None
MDOH-04-5M732^b^^,^^e^	Clinical	FL, United States	Unknown	3	3
F113A^b^	Food (clam)^c^	WA, United States	1988	36	36
MA561^b^^,^^e^	Food (oyster)	MA, United States	2016	631	None
G747^b^	Water	United States	2008	2021	None
G4186^b^	Food (oyster)	NH/ME, United States	Unknown	34	34
JBI17000682^d^	Clinical (feces)	FL, United States	2017	2666	None
JBI17000955^d^	Clinical (wound)	FL, United States	2017	154	None
JBI170001207^d^	Clinical (wound)	FL, United States	2017	1060	None
JBI17001588^d^	Clinical (feces)	FL, United States	2017	36	36

a FSL strains were provided by Martin Wiedmann, Cornell University, Department of Food Science (Yeung et al., 2002, 2003; Yeung and Boor, 2004). More information can be found on the Food Microbe Tracker Website: http://www.foodmicrobetracker.com/login/login.aspx

b Strains provided by Cheryl A. Whistler, University of New Hampshire (Mahoney et al., 2010; Ellis et al., 2012; Whistler et al., 2015; Xu et al., 2015, 2017a,b).

c Associated with an outbreak.

d Strains provided by Jamie DeMent, Florida Department of Health, Tallahassee, FL.

e Highlighted strains were used to amplify phages from water samples.

### Sequence typing of *Vibrio parahaemolyticus* isolates

STs of isolates were either characterized or confirmed by Multi Locus Sequence Typing (MLST) according to the standard method described by Jolley and Maiden (2010) and the database maintained by the University of Oxford and supported by the Wellcome Trust: https://pubmlst.org/organisms/vibrio-parahaemolyticus/ (Mahoney et al., 2010; Ellis et al., 2012; Whistler et al., 2015; Xu et al., 2015, 2017a,b). Chromosomal DNA was purified from each isolate, and seven genes were amplified by PCR using primers listed on the website indicated above. PCR products were purified and submitted to the Cornell genomics facility for sequencing. Sequence results were submitted to the online database to identify each bacterial isolate ST. Results are listed in Supplementary Table S2.

### Phage isolation

Phage were isolated from water samples collected along the North and South rims of Long Island, New York, in July 2017 (Supplementary Table S1). Salinity was measured with an American Marine Pinpoint Conductivity Monitor: conversion from μSiemens to ppt was performed as follows according to instructions: [(μSiemens/33) * 17.9]/1,000. All water samples were filtered through an 8 μm filter to eliminate large debris. Some of the water samples were subsequently filtered (0.22 μm) before use (samples indicated in phage name with an F for filtered with 0.22 μm and U for unfiltered). All samples were stored in the dark at 4°C. pH was measured with a Corning pH meter 430 equipped with an automatic temperature compensation (ATC) electrode. Not all water samples collected produced phage.

*Vibrio parahaemolyticus* infectious phages that were present in the collected coastal water samples were enriched as follows. For each water sample, 1 ml of 10X Tryptic Soy Broth (TSB) was diluted with 9 ml of coastal water and supplemented with 10 mM MgSO_4_. The broth was inoculated with seven isolates of *V. parahaemolyticus*: Strain ID# FSL Y1-012, FSL Y1-017, FSL Y1-036, FSL Y1-078, MDOH-04-5M732, F113A, and MA561 listed in Table 1, representing six STs. The seven isolates were selected for inoculation and incubation together. Isolates selected for the study included STs involved in outbreaks, and isolates from both sporadic clinical cases and food sources. Cultures were incubated at 30°C, 200 rpm overnight. Overnight cultures were treated with 500 μl of chloroform at 30°C for 30 min. Supernatants containing phages were cleared by centrifugation and stored at 4°C.

Individual phages were isolated using a plaquing assay. Isolates of *V. parahaemolyticus* were grown at 30°C 200 rpm to log phase in TSB-NaCl. Each *V. parahaemolyticus* isolate (200 μl) was mixed with phage supernatant (200 μl) and 10 mM MgSO_4_, then incubated at 30°C 20 min. Soft agar overlays were prepared by mixing 3.2 ml of melted Tryptic Soy Soft Agar (TSSA has 0.75% agar) + 2% NaCl at 55°C, with 200 μl of bacteria/phage reaction, and 10 mM MgSO_4_. The melted overlay was briefly vortexed and poured over a plate of TSA-NaCl. After an overnight incubation at 30°C, overlays were examined for the presence of plaques. Plaques were picked with a sterile tip and suspended in a small volume of SM buffer (100 mM NaCl, 8 mM MgSO_4_, 50 mM Tris–HCl pH 7.5, 0.01% gelatin) and treated with chloroform. Each phage was further purified by 3–4 rounds of plaquing and re-isolation of single plaques from overlays. In order to decrease the possibility of isolating sibling phages, a maximum of three phenotypically different plaques were picked per water sample.

### Characterization of phage infectivity range

Isolated phages were tested for plaquing with the 29 *V. parahaemolyticus* isolates and with *E. coli*, *B. subtilis*, *P. fluorescens*, *P. aeruginosa*, *E. piscicida*, *V. cholerae*, and *A. hydrophila* (to demonstrate bacteriophage species specificity). Bacterial overlays in soft agar were prepared as follows. Bacteria were grown overnight at 30°C 200 rpm. After overnight growth, bacteria were diluted and grown again in broth to exponential phase. 135 μl of a bacterial isolate grown in broth to exponential phase was mixed with 3.2 ml of melted TSSA +2% NaCl at 55°C. Serial dilutions of phages were spotted on the solidified agar (2 μl per spot) and incubated overnight at 30°C. Overlays were examined for the formation of individual plaques.

### CsCl phage purification

*Vibrio parahaemolyticus* FSL Y1-078 was cultured in 500 ml of TSB-NaCl supplemented with 10 mM MgSO_4_ to an OD_600_ of 0.2 and infected with a specific phage (27Ua.3, 29Fa.3, 31Fb.4, or 33Fb.4) at a MOI of ≈10. After an overnight incubation at 30°C 200 rpm, 5 ml of chloroform were added, and the incubation was continued for 10 min. RNAse and DNAse were added to a concentration of 1 μg/ml. NaCl was added to reach a final concentration of 1 M, followed by a 1-h incubation on ice. Particulates were pelleted at 15,000 *g* for 20 min at 4°C and the supernatant was decanted. Phages were precipitated from the supernatant by the gradual addition of 50 g of PEG8000 and incubated for a minimum of 2 h on ice water. Phages were pelleted at 11,000*g* for 15 min at 4°C and resuspended in 8 ml of SM Buffer. An equal volume of chloroform was added to extract PEG and the suspension was centrifuged at 3,000*g* for 15 min at 4°C. The aqueous layer was recovered, and the chloroform extraction was repeated until the PEG was all extracted. Phage volume was brought up to 10 ml with SM buffer and mixed with CsCl to a final concentration of 0.78 g/ml The suspension was centrifuged at 225,000*g* for 24 h at 4°C using a swinging bucket rotor. The band of purified phage was recovered, and the phage suspension was dialyzed against SM at 4°C. Purified phage was stored at 4°C. Phage titer was determined by spotting serial dilutions on an overlay of *V. parahaemolyticus* FSL Y1-078.

### Phage sequencing

Genomic DNA was purified from CsCl-purified phages using Zymo Research Viral DNA kit. The samples were submitted for whole genome sequencing to the Molecular Diagnostics Laboratory of the Animal Health Diagnostic Center of Cornell University. The libraries were constructed with Nextera XT DNA Library Prep with custom barcodes, random PCR (GenBank accession numbers in Supplementary Table S3).

### Genomic and phylogenetic analysis

Open reading frame (ORF) calling and preliminary annotation for each assembled phage genome was performed using pharokka (v1.0.0) with default parameters (Laslett and Canback, 2004; Chen et al., 2005; Bland et al., 2007; Steinegger and Soding, 2017; McNair et al., 2019; Alcock et al., 2020; Chan et al., 2021; Terzian et al., 2021). For phylogenetic comparisons of the major capsid and major tail proteins, seven publicly available *V. parahaemolyticus* phage sequences were downloaded from NCBI (Supplementary Table S3; Seguritan et al., 2003; Alanis Villa et al., 2012; Kim et al., 2012; Yuan et al., 2014; Lal and Ransangan, 2015; Pan et al., 2020). For consistent annotations, ORFs for major capsid and major tail genes were predicted using PhANNs with default parameters (Cantu et al., 2020). Major capsid and major tail sequences were identified as the highest scoring ORF, with median (±SE) PhANNs scores of 7.7 ± 0.2 and 5.9 ± 0.1, respectively, which correspond to reported confidence levels of ~95% and 85%, respectively. One previously published phage, VP16T, did not have an ORF score above 2.5 (~80% confidence) for the major tail protein and was dropped from that analysis. Phylogenetic analyses were performed using MEGA11 (version 11.0.11; Tamura et al., 2021): amino acid alignments for the PhANNs-identified ORFs for each protein were generated using MUSCLE, and then used to construct neighbor-joining trees with 1,000 bootstrap replicates.

### Electron microscopy

CsCl-purified phages were visualized by transmission electron microscopy (TEM). Samples were deposited onto a copper, 200 mesh, formvar and carbon coated grid and stained with 2% aqueous uranyl acetate. Samples were viewed on an FEI Tecnai 12 Biotwin transmission electron microscope. Images were taken with a high-resolution, high-contrast, thermoelectrically (TE) cooled Gatan Orius^®^ 1000 dual-scan CCD camera. It acquires 4,008 × 2,672 digital images, using Digital Micrograph (DM) software.

## Results

### Isolation of phages from coastal waters

Phages that were infectious for *V. parahaemolyticus* were isolated from water samples collected on July 31, 2017, at seven GPS locations along the North and South shores of Long Island, New York (Supplementary Table S1). Water parameters were as follows: temperature of 24°C–28°C, salinity of 21.30–24.20 ppt, and pH 7.5 to 8.3. Phages were identified by the formation of plaques in *V. parahaemolyticus* soft agar. The purified phages were stored in SM buffer at 4°C and amplified as needed in broth cultures of *V. parahaemolyticus*.

### Determination of phage infectivity range

The purified phages were initially tested against a collection of 29 *V. parahaemolyticus* isolates. Four phages, each one from a different water sample, were retained for their unique pattern of infectivity: 27Ua.3, 29Fa.3, 31Fb.4, and 33Fb.4 (Table 2). These four selected phages were infectious for subsets of seven to 12 *V. parahaemolyticus* isolates as determined by the formation of plaques on *V. parahaemolyticus* soft agar. A total of 17 *V. parahaemolyticus* strains were susceptible to at least one of the phages, whereas 12 *V. parahaemolyticus* strains were resistant to the four selected phages. The other phages not selected for further analyses either showed the same infectivity pattern as one of the four selected phages or inconsistency in infection. None of the other bacterial species tested (*E. coli*, *B. subtilis*, *P. fluorescens*, *P. aeruginosa*, *E. piscicida*, *V. cholerae*, and *A. hydrophila*) were susceptible to the four selected phages.

**Table 2:** Susceptibility patterns of four unique phages.

Strain ID	ST	Phage ID			
		27Ua.3	29Fa.3	31Fb.4	33Fb.4
FSL Y1-003	88	**−**	**+**	**−**	**−**
FSL Y1-005	8	**−**	**−**	**−**	**−**
FSL Y1-010	326	**−**	**+**	**−**	**−**
FSL Y1-012	1748	**−**	**−**	**−**	**+**
FSL Y1-013	54	**+**	**−**	**+**	**+**
FSL Y1-015	3	**−**	**−**	**−**	**−**
FSL Y1-016	3	**−**	**−**	**−**	**−**
FSL Y1-017	1464	**−**	**−**	**−**	**−**
FSL Y1-021	87	**−**	**−**	**−**	**−**
FSL Y1-023	3	**−**	**−**	**−**	**−**
FSL Y1-024	3	**−**	**−**	**−**	**−**
FSL Y1-025	3	**−**	**−**	**−**	**−**
FSL Y1-026	3	**−**	**−**	**−**	**−**
FSL Y1-036	3	**−**	**−**	**−**	**−**
FSL Y1-046	3	**−**	**−**	**−**	**−**
FSL Y1-059	676	**−**	**+**	**−**	**−**
FSL Y1-068	54	**−**	**−**	**+**	**+**
FSL Y1-069	26	**−**	**+**	**−**	**−**
FSL Y1-078	46	**+**	**+**	**+**	**+**
FSL Y1-079	46	**+**	**+**	**+**	**+**
MDOH-04-5 M732	3	**−**	**−**	**−**	**−**
F113A	36	**+**	**+**	**+**	**+**
MA561	631	**+**	**−**	**+**	**+**
G747	2021	**−**	**−**	**+**	**+**
G4186	34	**−**	**+**	**+**	**+**
JBI17000682	2666	**+**	**+**	**−**	**−**
JBI17000955	154	**−**	**−**	**−**	**+**
JBI170001207	1060	**−**	**−**	**+**	**+**
JBI17001588	36	**+**	**+**	**+**	**+**

To gain a better understanding of the infectivity of 27Ua.3, 29Fa.3, 31Fb.4, and 33Fb.4, the ST of each *V. parahaemolyticus* strain used in this study was determined (Table 1; Supplementary Table S2). The 29 *V. parahaemolyticus* strains represented 18 different STs that segregated into eight distinct patterns of susceptibility (PoS A-H; Table 3). Strains belonging to ST3 (CC3), ST1464 (CC3), ST8 (CC8), and ST87 were resistant to all four phages (PoS A), whereas strains belonging to ST36 (CC36) and ST46 were susceptible to all four phages (PoS H). Strains belonging to ST26 (CC24), ST88 (CC345), ST326, ST676, ST154, and ST1748 were susceptible to a single phage: 29Fa.3 (PoS B) or 33Fb.4 (PoS C). ST2666 and ST34 strains were the only representative of PoS D and PoS G, respectively. The two ST54 isolates were susceptible to different sets of phages (PoS E and PoS F): both isolates were susceptible to 31Fb.4 and 33Fb.4, but the second isolate was also susceptible to 27Ua.3 (PoS F). In addition, PoS F included the ST631 strain.

**Table 3:** Patterns of susceptibility of *Vibrio parahaemolyticus* ST and CC to phages.

Sequence type (ST)^a^	Clonal complex (CC)^b^	Pattern of Susceptibility (PoS)	Phage ID			
			27Ua.3	29Fa.3	31Fb.4	33Fb.4
3, 8, 87, 1464	3, 8	A	**−**	**−**	**−**	**−**
26, 88, 326, 676	24, 345	B	**−**	**+**	**−**	**−**
154, 1748		C	**−**	**−**	**−**	**+**
2666		D	**+**	**+**	**−**	**−**
54^c^, 1060, 2021		E	**−**	**−**	**+**	**+**
54^d^, 631		F	**+**	**−**	**+**	**+**
34	34	G	**−**	**+**	**+**	**+**
36, 46	36	H	**+**	**+**	**+**	**+**

a Underlined ST are from *V. parahaemolyticus* strains used to amplify phages from water samples (also indicated in Table 1).

b ST3 and ST1464 are part of CC3; ST8 is part of CC8; ST26 is part of CC24; ST88 is part of CC345; ST34 is part of CC34; ST36 is part of CC36; the other STs are not associated with CCs.

c Strain FSL Y1-068.

d Strain FSL Y1-013.

### Phage characterization by electron microscopy

**Figure 1:**
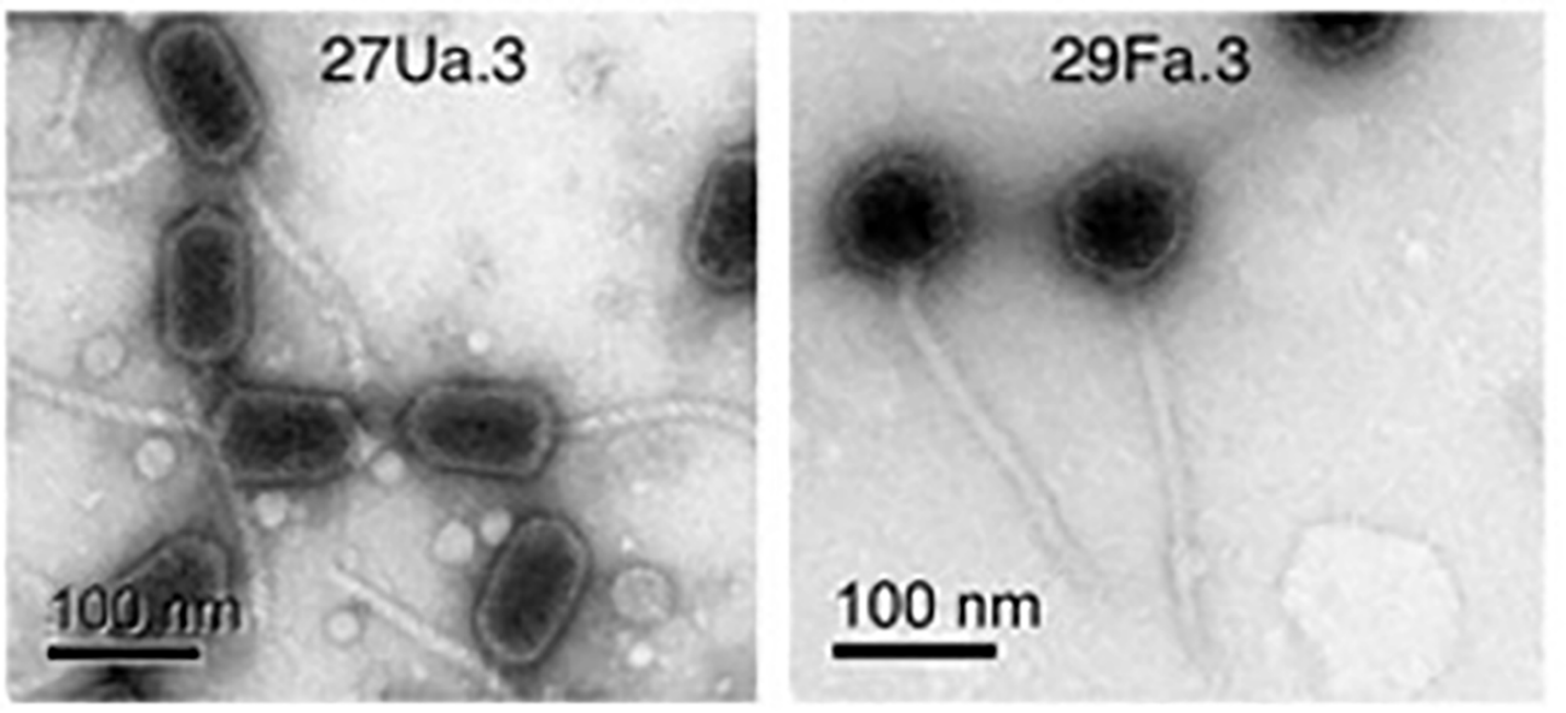
Representative purified phages were visualized by transmission electron microscopy. Left panel: 27Ua.3 showing elongated prolate heads with long tails (31Fb.4, and 33Fb.4 not shown had similar morphology). Right panel: 29Fa.3 showing icosahedral heads and long tails.

All four purified phages have icosahedral heads with long flexible non-contractile tails, characteristic of double-stranded DNA viruses of the *Siphoviridae* family (Figure 1). Head and tail measurements are reported in Table 4. Three of the phages, (27Ua.3, 31Fb.4, and 33Fb.4) have elongated prolate heads of 86–95 nm in length × 49–55 nm in width, with tails of 152–157 nm in length, whereas 29Fa.3 has a round head of 70 nm in diameter and a tail of 233 nm in length.

**Table 4:** Phage measurements from EM photographs.

	Phage ID			
	27Ua.3	29Fa.3	31Fb.4	33Fb.4
Head	91.21 ± 2.69^a^	70.40 ± 2.99	86.74 ± 3.97	95.02 ± 3.20
	54.87 ± 2.90^b^		49.12 ± 2.88	55.11 ± 2.07
	(12)^c^	(16)	(21)	(19)
Tail	157.35 ± 8.45	232.74 ± 11.36	154.18 ± 7.37	152.43 ± 10.99
	(12)	(16)	(21)	(19)

a Longitudinal measurements of phage heads and tails in nm ± standard deviation.

b Width measurements of phage heads in nm ± standard deviation.

c Number of measurements in parentheses.

### Phage sequences and phylogenetic analysis

The sequenced and assembled phages 27Ua.3, 29Fa.3, 31Fb.4, and 33Fb.4 each produced a single contig with high read coverage: genome sizes were 76.890, 79.348, 77.620, and 77.632 kb, with calculated GC contents of 48.8%, 46.8%, 48.9%, and 48.9%, respectively. Putative ORFs and preliminary annotations were predicted using pharokka (v1.0.0) with default parameters (Laslett and Canback, 2004; Chen et al., 2005; Bland et al., 2007; Steinegger and Soding, 2017; McNair et al., 2019; Alcock et al., 2020; Chan et al., 2021; Terzian et al., 2021).

**Figure 2:**
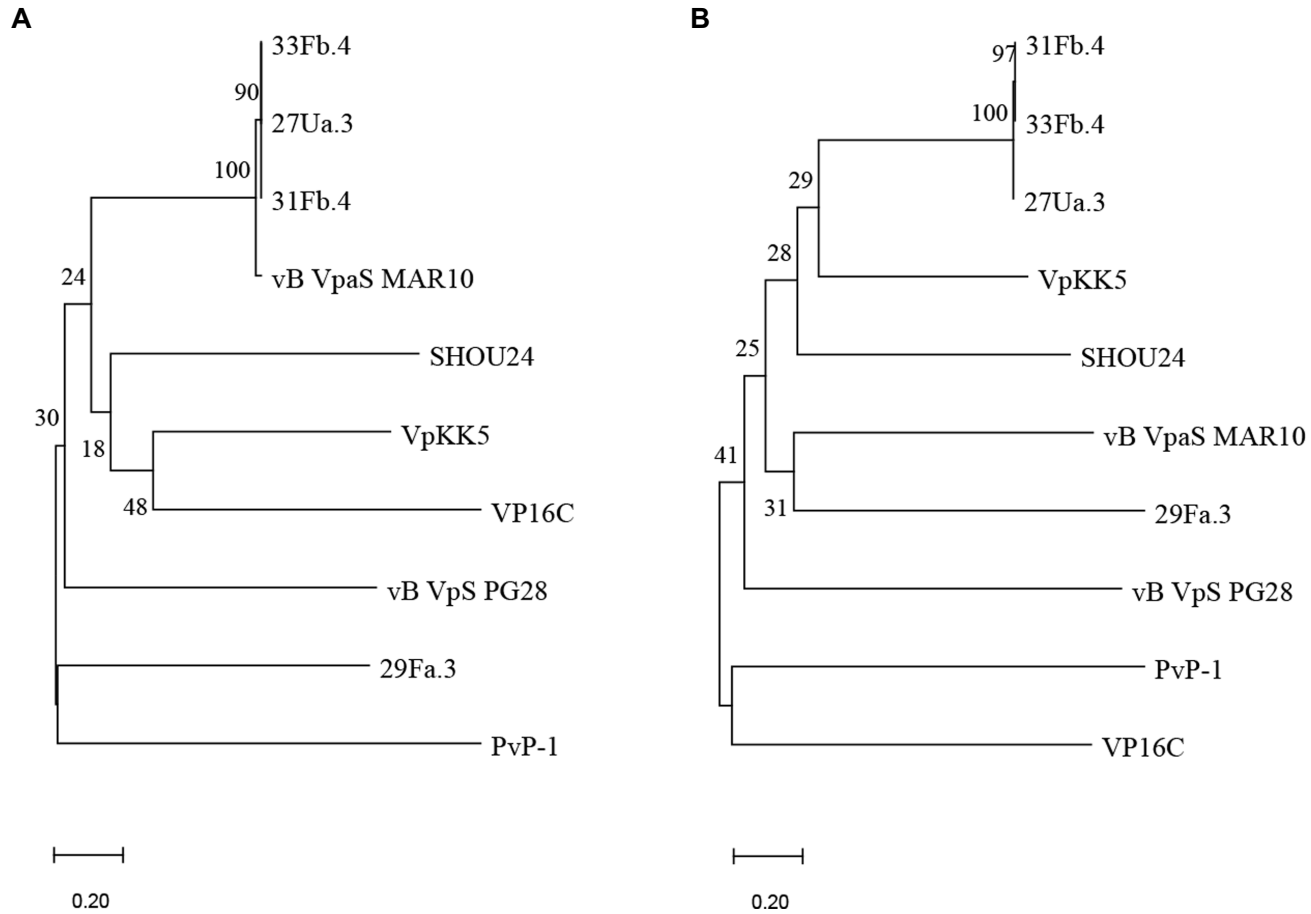
Phylogenetic trees based on the predicted major capsid **(A)** and major tail **(B)** proteins of the phages isolated in this study (27Ua.3, 29Fa.3, 31Fb.4, and 33Fb.4) and other publicly available *Vibrio parahaemolyticus* phages. Protein identities were predicted from ORFs or coding sequences using PhANNs, and analyzed using MEGA11: all putative amino acid sequences were aligned using MUSCLE and phylogenetic trees were constructed by neighbor-joining with 1,000 bootstrap replicates. Nodes are marked with the bootstrap value as percent.

To determine whether phylogenetic relationships between the isolated novel phage mirrored the infectivity patterns, we generated protein alignments of the predicted major capsid and major tail proteins of the four purified phages (27Ua.3, 29Fa.3, 31Fb.4, and 33Fb), as well as seven previously published *V. parahaemolyticus* phages (Supplementary Table S3). Because the novel and published phage genomes lacked annotation for many structural proteins, we first predicted structural protein ORFs for each using PhANNs (Cantu et al., 2020). Phylogenetic analysis of the PhANNs-predicted major capsid and major tail sequences reveal that phages 31Fb.4, 33Fb.4 and 27Ua.3 are closely related, and share major capsid gene similarity with phage MAR10 (Villa et al., 2012; Figure 2). Phage 29Fa.3 was more closely related to other phage included in the comparison, though its closest relative varied depending on the protein used for tree construction. These phylogenetic patterns parallel in part the phage infectivity profiles (Table 3), with 31Fb.4 and 33Fb.4 infecting nearly the same STs, and 29Fa.3 having the most distinct profile.

## Discussion

In this study, we explored the possibility that monitoring coastal waters for bacteriophages could facilitate early detection of *V. parahaemolyticus* STs potentially associated with seafood-borne illness. Using a selection of four bacteriophages enriched and isolated from New York Atlantic coastal waters and 29 strains of *V. parahaemolyticus* representing 18 STs, we observed that different STs were susceptible to different sets of phages. First, the nine isolates representing ST3 and the single ST1464 isolate were resistant to infection by all four phages. ST3 and ST1464 are part of CC3, which predominated seafood outbreaks in 1997–1998 (Nair et al., 2007), but have now been replaced by ST36 and ST631 in the United States (Haendiges et al., 2014; Xu et al., 2015, 2017a,b). This result suggests that *V. parahaemolyticus* belonging to CC3 were absent from coastal waters at the time of this study, as phage propagation is dependent on the presence of its specific bacterial host. On the other hand, ST36 was susceptible to all four phages isolated from the coastal waters, suggesting that ST36 likely populated the coastal waters at the time of collection. However, this susceptibility pattern was not restricted to ST36, as two ST46 isolates of unknown origin were also susceptible to all four phages. ST631 was susceptible to the same set of phages as one of the ST54 strains, which originated from a clinical case. Also noteworthy were patterns of susceptibility C and D, which included isolates of three different STs from sporadic clinical cases. In addition, an isolate from oysters (ST34 (NH/ME)) had a unique pattern of susceptibility (PoS G); ST34 has previously been associated with clinical cases (Miller et al., 2021).

A previous study showed that phages enriched from oysters out of Delaware Bay did not infect *V. parahaemolyticus* serotype O3:K6 (during specific collection periods), the serotype representing a previous pandemic strain (Richards et al., 2019). The present study included eight *V. parahaemolyticus* strains representing serotype O3:K6 (including FSL Y1-016, 017, 021, 023, 024, 025, 026, and 046; Yeung and Boor, 2004). These strains, which are part of CC3 with the exception of FSL Y1-021 (ST87), were resistant to infection by all four phages. Together, these results suggest that *V. parahaemolyticus* strains belonging to CC3 were absent from coastal waters at the time of these two independent studies, supporting the possibility that phages could be used to monitor the populations of *V. parahaemolyticus* STs that prevail around oyster farms at various times of the year. However, expanding the number of phage isolates and *V. parahaemolyticus* strains to identify greater inclusivity and specificity would be necessary for this approach to be useful as a prevention measure for potential food outbreaks.

The four phages isolated in this study (27Ua.3, 29Fa.3, 31Fb.4, and 33Fb.4) were sequenced and genome lengths and percent GC content values were compared to other vibriophage data available in the NCBI database. The previously published phages demonstrated characteristics of Siphoviruses with the exception of vBVpSPG28 showing tail morphology similar to that of Myoviruses (Tian et al., 2022). Phage genome lengths and percent GC content were similar to vB VpaS MAR10 (isolated off the coast of Mexico) and SHOU24 (isolated from aquatic market sewage in Shanghai, China; Villa et al., 2012; Yuan et al., 2014). Of the previously published phages, only vB VpaS MAR10 has an elongated head similar to those of 27Ua.3, 31Fb.4, and 33Fb.4 (Villa et al., 2012); the phylogenetic analysis of the major capsid protein of these four phages indicate that they are closely related. However, there is no information related to the infectivity of these other phages for the various STs representing *V. parahaemolyticus* strains.

*Vibrio parahaemolyticus* is a very diverse bacterial species. Some strains are human pathogens, others are pathogenic for aquatic animals, whereas many have no known pathogenic characteristics. Current diagnostic methods of *V. parahaemolyticus*, which require bacterial culture and biochemical, phenotypic, or molecular analyses can be time consuming and labor intensive. Newer molecular approaches for identification that are growing in use, including the 4 amplicon multiplex PCR detection assay designed to specifically identify ST36 isolates, have reduced the time to a result but are still a resource burden (Letchumanan et al., 2014; Whistler et al., 2015). To reduce costs and time to a result, the LAMP (loop-mediated isothermal amplification) test that is widely used to detect SARS-CoV2, was modified to detect *V. parahaemolyticus* in clinical samples (Zhou et al., 2021). Unfortunately, this test has limited accuracy. Thus, there is a need to develop additional methods that are cost efficient and specific for foodborne-related pathogenic strains of *V. parahaemolyticus*.

The present study informs future applications for use of bacteriophages in differentiating *V. parahaemolyticus* ST and screening for the presence of STs associated with foodborne illnesses.

Phages could be used to help monitor coastal waters in which shellfish are cultured. As phages are relatively easy to isolate and multiply exponentially when their specific hosts are abundant, newly isolated phages could be used as an initial proxy for detection of *V. parahaemolyticus* STs populating the waters. Considering that bacteria can acquire resistance to phages they encounter (Bondy-Denomy and Davidson, 2014), the battery of phages used to monitor waters will have to be constantly updated using *V. parahaemolyticus* isolates from recent infections. Moreover, large scale studies will be needed to assess statistical significance and correlation.

In conclusion, this study identified and sequenced four novel phages infectious for 18 distinct STs of *V. parahaemolyticus*. Phylogenetic analyses suggest that three of these novel phages form a new clade distinct from previously sequenced and published *V. parahaemolyticus* phage. The distinct phage susceptibility patterns of the various *V. parahaemolyticus* STs support the potential use of phages as a means for monitoring contamination of waters and shellfish. Pre-harvest detection of *V. parahaemolyticus* STs of interest would contribute to decreased morbidity and mortality due to foodborne infections, increase consumer confidence in the safety of shellfish, and consequently increase the profitability and sustainability of the industry.

## Data availability statement

The data presented in the study are deposited in GenBank with accession numbers OP547477, OP547478, OP595601, OP595602.

## Author contributions

KBS: study design, sample collection, data acquisition, analyses, interpretation, manuscript writing, manuscript revision. JR: sample collection, data acquisition, analyses, manuscript writing, manuscript revision. RS-C: data acquisition, analyses, interpretation, manuscript writing, manuscript revision. KLS: sample collection, data acquisition, analyses, manuscript revision. SC: data acquisition, analyses, manuscript writing, manuscript revision. MH: data acquisition, manuscript revision. GR: study review, sample collection, manuscript revision, CW and MW: study review, strain supply, manuscript revision. SJ: study review, manuscript revision. JD: strain supply, manuscript revision. RG: study review, laboratory consultation, manuscript revision. HM: study conception, study design, sample collection, analyses, interpretation, manuscript writing, manuscript revision. All authors contributed to the article and approved the submitted version.

## Funding

This project was funded by the Northeastern Regional Aquaculture Center Award No. 2016-38500-25754 and Cornell Center for Materials Research Shared Facilities which are supported through the NSF MRSEC program (DMR-1719875).

## Conflict of interest

The authors declare that the research was conducted in the absence of any commercial or financial relationships that could be construed as a potential conflict of interest.

## Publisher’s note

All claims expressed in this article are solely those of the authors and do not necessarily represent those of their affiliated organizations, or those of the publisher, the editors and the reviewers. Any product that may be evaluated in this article, or claim that may be made by its manufacturer, is not guaranteed or endorsed by the publisher.

## Footnotes

^1^ https://wwwn.cdc.gov/norsdashboard/

^2^ https://www.fda.gov/food/federalstate-food-programs/national-shellfish-sanitation-program-nssp
