# Supplementary material for: Coastal water bacteriophages infect various sets of Vibrio parahaemolyticus sequence types
Source: Front Microbiol. 2022 Dec 19;13:1041942. doi: 10.3389/fmicb.2022.1041942 (PMC9807174; doi:10.3389/fmicb.2022.1041942)
Supplement: Supplementary file 1 [file Data_Sheet_1.zip › Supplementary_MaterialFrontiers.pdf]

Supplementary Material

1 Supplementary Tables

Table S1 Water samples collected on Long Island, New York (July 31, 2017)

Collection Data		Water Parameters			
Location	GPS Parameters	pH	Salinity (ppt)	Temp (C°)	Phages Isolated [a]
Oyster Bay	40°54’14”N 73°33’4”W	7.6	21.51	28	27Ua.3
Oyster Bay	40°54’18”N 3°33’22”W	7.5	21.51	26	29Fa.3
Port Jefferson	40°53’53”N 73°26’6”W	8.0	21.30	24	31Fb.4
Fire Island National Seashore	40°39’15” N 73°14’6”W	8.3	24.20	27	33Fb.4

[a]Phage identification key: water collection number (27-33); unfiltered (U) or filtered (F); plaques isolated per water sample (a and b); number of plaque re-isolation steps (3 or 4)

Table S2 Multi Locus Sequence Typing results for *V. parahaemolyticus* isolates

MARQ ID	Sequence Type (ST)	Clonal Complex (CC)	Gene						
			dnaE	*gyrB*	*recA*	*dtdS*	*pntA*	*pyrC*	*tnaA*
FSL Y1-003	88	345	(11)[a]	48	43	48	26	39	26
FSL Y1-005	8	8	28	4	82	88	23	69	(47)
FSL Y1-010	326	-	N.D.[b]	28	44	46	61	49	38
FSL Y1-012	1748	-	361	489	30	29	26	22	54
FSL Y1-013	54	-	31	8	(22)	13	N.D	7	2
FSL Y1-015	3	3	3	4	19	4	29	4	22
FSL Y1-016	3	3	3	4	19	4	29	4	22
FSL Y1-017	1464	3	62	4	19	4	29	4	22
FSL Y1-021	87	-	11	4	16	35	29	52	22
FSL Y1-023	3	3	3	4	19	4	29	4	22
FSL Y1-024	3	3	3	4	19	4	29	4	22
FSL Y1-025	3	3	3	4	19	4	29	4	22
FSL Y1-026	3	3	3	4	19	4	29	4	22
FSL Y1-036	3	3	3	4	19	4	29	4	22
FSL Y1-046	3	3	3	4	19	4	29	4	22
FSL Y1-059	676	-	60	N.D.	31	72	66	62	65
FSL Y1-068	54	-	31	8	N.D.	13	30	7	2
FSL Y1-069	26	24	17	19	13	36	(26)	32	26
FSL Y1-078	46	-	25	35	4	8	33	31	6
FSL Y1-079	46	-	25	35	4	8	33	31	6
MDOH-04-5M732	3[c]	3	N.D.	4	19	4	29	4	22
F113A	36[c]	36	21	15	1	23	23	21	16
MA561	631[c]	-	222	128	21	69	(54)	236	12
G747	2021	-	6	414	384	206	46	192	20
G4186	34[c]	34							
JBI17000682	2666	-	221	298	258	206	78	14	87
JBI17000955	154	-	35	50	63	27	49	46	26
JBI170001207	1060	-	224	90	273	19	(26)	238	68
JBI17001588	36	36	21	15	1	23	23	21	16

[a] Parentheses indicate that the allele number does not match the number provided by the *V. parahaemolyticus* public database (https://pubmlst.org/vparahaemolyticus/).
[b] N.D.: Allele number Not Determined. Forward and reverse sequences result in different allele number.
[C] Previously sequenced typed (5, 9, 12). Allele numbers are indicated for the strains that were sequenced typed as a control.

Table S3 *Vibrio parahaemolyticus* phages

Name	Accession number	Genome length (bp)	GC content (%)	Reference
27Ua.3	OP547477	76,890	48.8	this study
29Fa.3	OP547478	79,348	46.8	this study
31Fb.4	OP595601	77,620	48.9	this study
33Fb.4	OP595602	77,632	48.9	this study
VpKK5	NC_026610.2	56,637	51.3	(1)
SHOU24	NC_023569.1	77,837	46.0	(2)
vB VpaS MAR10	NC_019713.1	78,751	49.7	(3)
pVp-1	NC_019529.1	111,506	39.7	(4)
VP16T [a]	AY328852.1	49,575	58.8	(5)
VP16C [a]	AY328853.1	47,537	59.4	(5)
vB VpS PG28	MT735630.2	82,712	48.1	(6)

1. Lal TM, Ransangan J. 2015. Complete Genome Sequence of VpKK5, a Novel Vibrio parahaemolyticus Lytic Siphophage. Genome Announc 3.
2. Yuan L, Cui Z, Wang Y, Guo X, Zhao Y. 2014. Complete genome sequence of virulent bacteriophage SHOU24, which infects foodborne pathogenic Vibrio parahaemolyticus. Arch Virol 159:3089–93.
3. Alanis Villa A, Kropinski AM, Abbasifar R, Griffiths MW. 2012. Complete genome sequence of Vibrio parahaemolyticus bacteriophage vB_VpaM_MAR. J Virol 86:13138–9.

4. Kim JH, Jun JW, Choresca CH, Shin SP, Han JE, Park SC. 2012. Complete genome sequence of a novel marine siphovirus, pVp–1, infecting Vibrio parahaemolyticus. J Virol 86:7013–4.
5. Seguritan V, Feng IW, Rohwer F, Swift M, Segall AM. 2003. Genome sequences of two closely related Vibrio parahaemolyticus phages, VP16T and VP16C. J Bacteriol 185:6434–47.
6. Pan Q, Ren H, Sun H, Tong Y, Yan Y, Zhao F. 2020. Vibrio phage vB_VpS_PG28, complete genome [GenBank: MT735630.2]. NCBI.
