# Supplementary material for: Coastal water bacteriophages infect various sets of Vibrio parahaemolyticus sequence types
Source: Front Microbiol. 2022 Dec 19;13:1041942. doi: 10.3389/fmicb.2022.1041942 (PMC9807174; doi:10.3389/fmicb.2022.1041942)

## 1 Figures

**Figure 1**. Representative purified phages were visualized by transmission electron microscopy. Left panel: 27Ua.3 showing elongated prolate heads with long tails (31Fb.4, and 33Fb.4 not shown had similar morphology). Right panel: 29Fa.3 showing icosahedral heads and long tails.

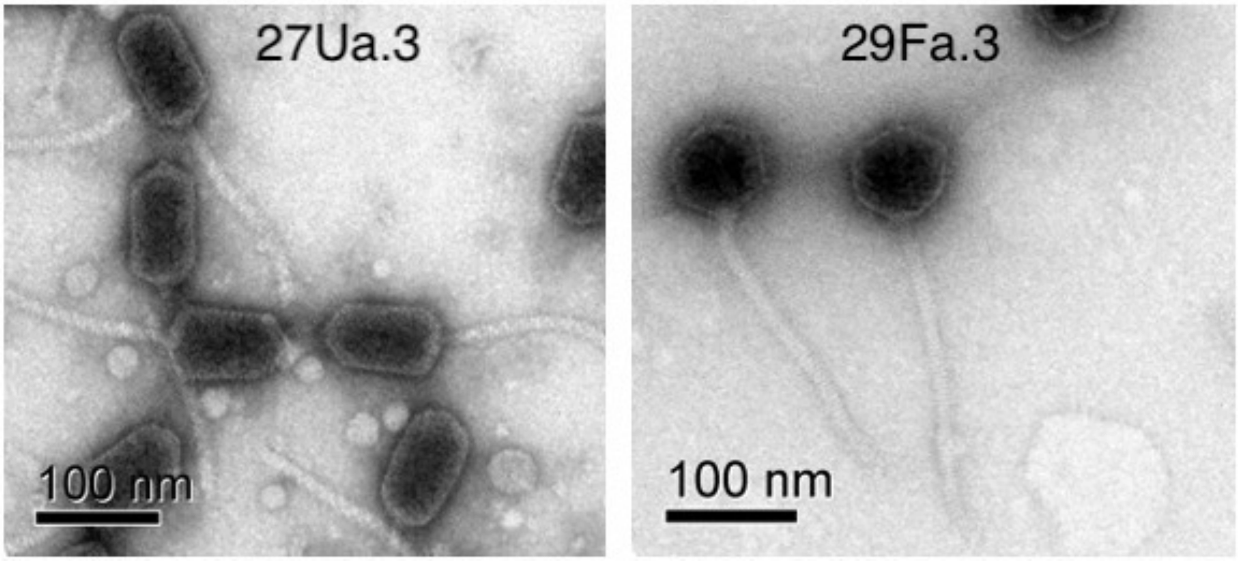

Supplement: Supplementary file 1 [file Data_Sheet_1.zip › Figure 1.pdf]
