# Supplementary material for: Coastal water bacteriophages infect various sets of Vibrio parahaemolyticus sequence types
Source: Front Microbiol. 2022 Dec 19;13:1041942. doi: 10.3389/fmicb.2022.1041942 (PMC9807174; doi:10.3389/fmicb.2022.1041942)

1 Figures

Figure 2. Phylogenetic trees based on the predicted major capsid (**A**) and major tail (**B**) proteins of the phages isolated in this study (27Ua.3, 29Fa.3, 31Fb.4, and 33Fb.4) and other publicly available *V. parahaemolyticus* phages. Protein identities were predicted from ORFs or coding sequences using PhANNs, and analyzed using MEGA11: all putative amino acid sequences were aligned using MUSCLE and phylogenetic trees were constructed by neighbor-joining with 1,000 bootstrap replicates. Nodes are marked with the bootstrap value as percent.

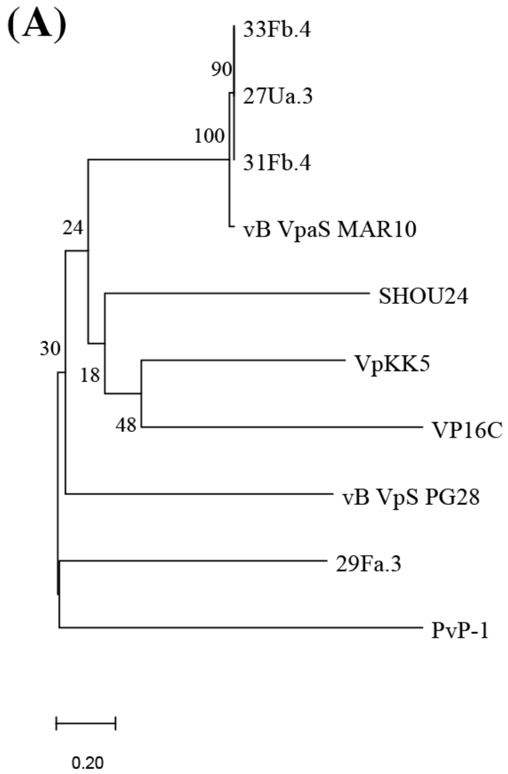

Supplement: Supplementary file 1 [file Data_Sheet_1.zip › Figure2.pdf]
